# Low pH of interstitial fluid around hippocampus of the brain in diabetic OLETF rats

**DOI:** 10.1186/2052-8426-2-6

**Published:** 2014-03-01

**Authors:** Yoshinori Marunaka, Kanji Yoshimoto, Wataru Aoi, Shigekuni Hosogi, Hiroshi Ikegaya

**Affiliations:** Department of Molecular Cell Physiology, Graduate School of Medical Science, Kyoto Prefectural University of Medicine, Kyoto, 602-8566 Japan; Department of Bio-Ionomics, Graduate School of Medical Science, Kyoto Prefectural University of Medicine, Kyoto, 602-8566 Japan; Department of Forensic Medicine, Graduate School of Medical Science, Kyoto Prefectural University of Medicine, Kyoto, 602-8566 Japan; Departments of Food Sciences and Biotechnology, and Health, Faculty of Life Sciences, Hiroshima Institute of Technology, Hiroshima, 731-5193 Japan; Laboratory of Health Science, Graduate School of Life and Environmental Sciences, Kyoto Prefectural University, Kyoto, 606-8522 Japan; Japan Institute for Food Education and Health, St. Agnes’ University, Kyoto, 602-8013 Japan

**Keywords:** pH, Hippocampus, Diabetes mellitus, OLETF rat, Antimony pH electrode

## Abstract

**Background:**

We have reported that pH values of ascites and interstitial fluids around the liver in Otsuka Long-Evans Tokushima Fatty (OLETF) rats are significantly lower than normal pH, 7.40, of mammalian body fluids (Biochem Biophys Res Commun 2013, 432:650), and that this lowered pH of interstitial fluid causes the insulin resistance in diabetic patients by decreasing insulin-binding to its receptors (J Physiol Sci 2013, 63:S199). In the preset study, we tried to measure the interstitial fluid pH in diabetic OLETF rats, since the interstitial fluid pH plays key factors in the brain function from a viewpoint of the binding affinity of neurotransmitters to their receptors.

**Findings:**

We found that the pH value of interstitial fluids around hippocampus, the most important area for memory, in diabetic OLETF rats was lower than that in normal rats by measuring pH with antimony pH electrodes.

**Conclusions:**

The lowered pH of interstitial fluid around hippocampus of the brain in diabetic rats observed in the present study suggests that the function of hippocampus of the brain would be diminished due to low affinity of various types of neurotransmitters, playing key roles in the hippocampus function, to their receptors. Therefore, we indicate that maintenance of the interstitial fluid pH at the normal level would be one of the most important key factors for molecular and cellular therapies in various types of diseases including diabetes mellitus.

## Findings

Metabolic syndromes called for common diseases based on metabolic disorders are developing worldwide, referring to visceral obesity, high blood glucose levels, dyslipidemia and hypertension. Further, metabolic syndromes lead to an increase in the risk of developing of diabetes mellitus (type 2). As shown in our previous report [1], pH of ascites and interstitial fluids around the liver in OLETF rats is significantly lower than normal pH, 7.40, of mammalian body fluids. Further, our report indicates that insulin resistance occurs associated with lowered pH of interstitial fluid [2]. Insulin resistance has been reported to play a critical role in development of cardiovascular disease; *i.e.*, insulin sensitivity is closely associated with blood pressure [3–6]. The lowered pH of interstitial fluid, which causes insulin resistance [2], would play important roles in various types of diseases including dysfunction of neural cells in the brain including dementia frequently appearing in diabetes mellitus patients.

### Research hypothesis

Based on the information described above, we hypothesize that the interstitial fluid pH around hippocampus of the brain in OLETF rats is lower than that in normal rats.

### Materials

Six-week-old Otsuka Long-Evans Tokushima Fatty (OLETF) and Wistar rats were obtained from SHIMIZU Laboratory Supplies Co., Ltd. (Kyoto, Japan), and were maintained for 20 weeks (up to 26-week-old) similar to reported in our previous report [1, 7].

### Measurement of the interstitial fluid pH

We measured the interstitial fluid pH around hippocampus of the 26-week-old rat brain using antimony pH electrodes (Chemical Instruments, Hachioji, Tokyo, Japan) [8], approaching the interstitial area around hippocampus of the rat brain with a method described in our previous report [9]. The approach of pH electrodes might produce a small space around hippocampus of the brain, into which a small amount of cerebrospinal fluids might move. Therefore, the pH value shown in the present study might indicate that of the interstitial fluid containing a small amount of cerebrospinal fluid, although both fluids are extracellular ones and movements of cerebrospinal fluid have no influence on measured pH values. In the present study, we show pH values at 60 and 90 min after pH electrodes reached interstitial fluids around hippocampus of the brain in OLETF and normal (Wistar) rats, since we obtained steady pH values 60 min after pH electrodes reached the brain area.

### Data presentation and statistical analysis

The pH value is shown as the mean, and the error bar indicates SEM. Student *t*-test was applied to determine significance of difference between pH values of OLETF and normal rats.

### Ethics

We performed all animal studies in accordance with the guidelines of the Japanese Council on Animal Care and approved by the Committee for Animal Research of Kyoto Prefectural University of Medicine.

## Results

**Figure 1:**
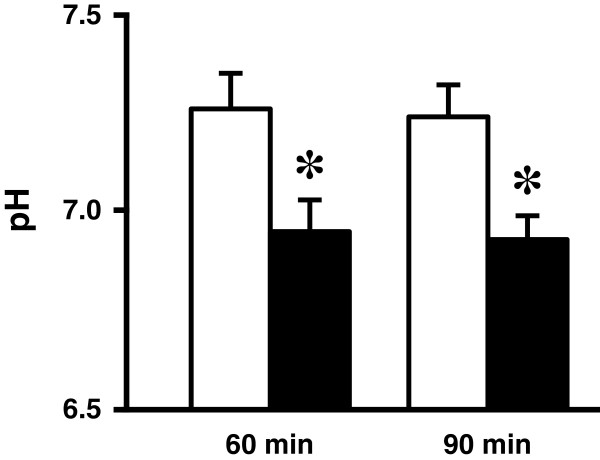
**pH of interstitial (extracellular) fluid around hippocampus of OLETF and normal (Wistar) ratsv.** The pH value is shown as the mean, and the error bar indicates SEM. The pH values shown in Figure 1 were measured at 60 and 90 min after antimony pH electrodes reached interstitial (extracellular) fluids around hippocampus of the OLETF rats (closed columns) and normal (Wistar) rats (open columns). n = 4. *, *p* < 0.05 compared with that in normal (Wistar) rats at each measured time.

We found that pH of interstitial fluids around hippocampus of the brain, the most important area for memory, in diabetic OLETF rats was lower than that in normal (Wistar) rats under steady state conditions as shown in Figure 1, which indicates pH values at 60 and 90 min after antimony pH electrodes reached the interstitial fluid around hippocampus of the brain of OLETF and normal (Wistar) rats.

## Discussion

**Figure 2:**
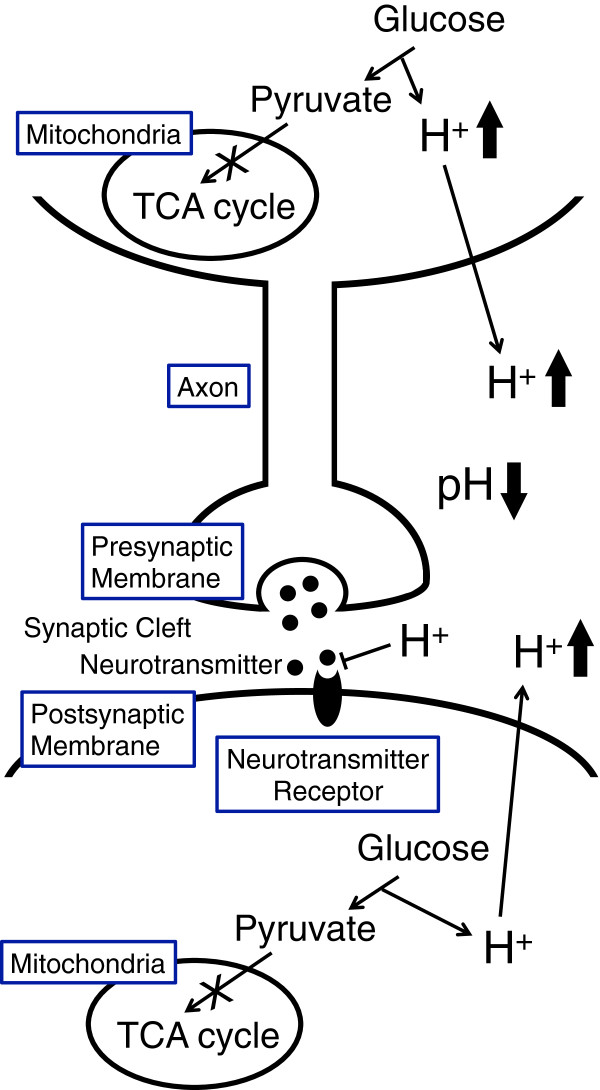
**The potential molecular mechanism of pH-dependent regulation of neural cell function in diabetes mellitus with mitochondria dysfunction.** Neural cells with mitochondria dysfunction synthesize ATP required for maintenance of neural cell function only or mainly via glycolysis, producing much larger amounts of H^+^ than neural cells with normal function of mitochondria synthesizing ATP via TCA cycle. This large amount of H^+^ produced by glycolysis in neural cells with dysfunction of mitochondria is released to the extracellular space (interstitial fluid) including the synaptic cleft. This phenomenon results in lowered pH of the extracellular space (interstitial fluid) including the synaptic cleft. Activity of neural cells at lowered pH of the extracellular space (interstitial fluid) is kept low due to a low level of synaptic neurotransmission signals via low binding affinity of neurotransmitters to their receptors. Namely, the amount of neurotransmitters released into the synaptic cleft, which is large enough for generation of action potential under conditions with normal function of mitochondria, is insufficient for generating action potential under lowered pH conditions due to low binding affinity of neurotransmitters to their receptors.

Regulation of ion circumstances plays important roles in various cell functions [10–19]. The present report clearly indicates that pH of interstitial fluid around hippocampus of the brain of the diabetic OLETF rats, whose pH of ascites and interstitial fluids around liver has been reported to be low [1], was significantly lower than normal (Wistar) non-diabetic rats. Under a condition with diabetes mellitus, mitochondria dysfunction has been reported [20]. Dysfunction of mitochondria leads cells to synthesis of ATP required for maintenance of neural cell function only or mainly by glycolysis, which produces much larger amounts of H^+^ compared with that produced by mitochondria (TCA cycle)-based ATP synthesis in neural cells with normal mitochondria function. This large amount of H^+^ produced by glycolysis under a condition with dysfunction of mitochondria in diabetes mellitus is released to the extracellular space (interstitial fluid) including the synaptic cleft via various types of ion transporter [10], resulting in lowered pH of the extracellular space (interstitial fluid) including the synaptic cleft (Figure 2).

Lowered pH of the interstitial fluid in diabetic OLETF rats [1], which diminishes the binding affinity of insulin to its receptor, causes the insulin resistance [2]. In neural cells, pH of the interstitial (extracellular) fluid plays key roles in regulation of neurotransmitters/hormones binding affinity to their receptors [21, 22]. Therefore, similar to lowered pH-induced insulin resistance in diabetic mellitus [1, 2], lowered pH of interstitial fluids around hippocampus of the brain shown in the present study would diminish the binding affinity of neurotransmitters to their receptors. This means that even if the amount of neurotransmitters released into the synaptic cleft is large enough under the normal pH condition for generation of action potential, this amount of neurotransmitters is not sufficient for generating action potential due to low binding affinity of neurotransmitters to their receptors.

Indeed, it has been reported that diabetes mellitus patients have a high risk in dementia including Alzheimer’s disease [23] with disturbance of memorizing function. Hippocampus is a key area in the brain playing a crucial role in function of memory [24]. Thus, these findings suggest that lowered pH of interstitial fluid around hippocampus of the brain in diabetes mellitus would be one of the main pathogeneses causing disturbance of memorizing function in some diseases such as Alzheimer’s disease.

Based on the information obtained in the present study and other reports mentioned above, we suggest that maintenance of the interstitial fluid pH at the normal level or recovery of the interstitial pH from lowered levels via application of compounds influencing the pH itself and/or pH buffer capacitance would be one of the most important key factors for molecular and cellular therapies in various types of metabolic disorders including diabetes mellitus. We should try to search compounds influencing the pH itself and/or pH buffer capacitance, and apply the compound to model animals with metabolic disorders including diabetes mellitus. After success in search and studies in animal models, we will try to perform human studies using those compounds.
